# Flavohaemoglobin: the pre-eminent nitric oxide–detoxifying machine of microorganisms

**DOI:** 10.12688/f1000research.20563.1

**Published:** 2020-01-08

**Authors:** Robert K. Poole

**Affiliations:** 1 Department of Molecular Biology and Biotechnology, The University of Sheffield, Firth Court, Sheffield, S10 2TN, UK

**Keywords:** flavohaemoglobin, nitric oxide, microbiology

## Abstract

Flavohaemoglobins were first described in yeast as early as the 1970s but their functions were unclear. The surge in interest in nitric oxide biology and both serendipitous and hypothesis-driven discoveries in bacterial systems have transformed our understanding of this unusual two-domain globin into a comprehensive, yet undoubtedly incomplete, appreciation of its pre-eminent role in nitric oxide detoxification. Here, I focus on research on the flavohaemoglobins of microorganisms, especially of bacteria, and update several earlier and more comprehensive reviews, emphasising advances over the past 5 to 10 years and some controversies that have arisen. Inevitably, in light of space restrictions, details of nitric oxide metabolism and globins in higher organisms are brief.

## Nitric oxide in biology and a caveat

**Figure 1.**
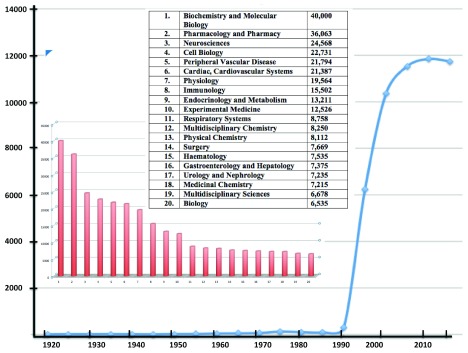
Publications per annum found by using the search term “nitric AND oxide” in Web of Science Core Collection, December 2019. The inset shows the number of citations in the search categorised by subject area.

The importance of the small gas nitric oxide (NO) can hardly have escaped the attention of most scientists in the fields of clinical medicine, physiology, biochemistry, microbiology and environmental science. “Popular” textbooks describe this science and medicine ^1, 2^. Total citations of “nitric” AND “oxide” in the Web of Science Core Collection approach 300,000. Until the past few decades, the literature was dominated by chemistry but now the largest category is biochemistry and molecular biology (>42,000 citations, representing more than 14% of the total; Figure 1). This clearly demonstrates the pervasive impact of NO in biology. This short review aims to link these impacts with one of the several enzymes known to destroy this invaluable yet potentially toxic molecule.

NO is a free radical species; it may therefore be written formally as NO ^.^ (NO-dot) but conventionally simply as NO. The chemistry of NO is complex and, as a result, there are numerous intricacies, misunderstandings and sometimes errors in the literature. It is soluble in water (approximately 1.6 mM at 37 °C and 1.94 mM at 25 °C ^3^) but does not react with it. The difficulties stem from its short lifetime in cellular environments: if generated at 10 ^−7^ M (for example, from NO synthases, or NOSs), NO has a lifetime of 30 min if its fate is oxidation to NO _2_ but may be as low as 1 s on reaction with biological targets, especially haems, thiols and superoxide anion ^4^. A multitude of redox-related species may be generated from NO in biological situations and each may have different targets. This chemistry is not detailed here except where it is required for clarity, but excellent reviews exist ^5– 7^. In brief, nitrosonium cation (NO ^+^), nitroxyl anion (NO ^−^, although HNO is dominant at pH 7), nitrogen dioxide (NO _2_), and dinitrogen trioxide (N _2_O _3_, the product of NO reacting with O _2_) and peroxynitrite (ONOO ^−^, the product of NO reacting with superoxide radical) are not “forms of NO” but products of NO reactions. There is only one NO ^4, 8^!

The major source of NO *in vivo* is via the activity of NOSs. The complex biology, chemistry and medical significance of NOS are outside the scope of this commentary, but excellent reviews and articles cover mammalian ^9^, microbial ^10^ and the elusive plant NOS-like ^11^ activities. The roles of NO in signalling and “gasotransmitters” in higher organisms are also beyond the scope of this article, but see 7. In higher organisms, NO plays a key role in cellular immunity, where the gas, generated primarily by inducible NOS, attacks diverse macromolecules in invading microbes ^6^. The only organisms in which flavohaemoglobins are found are microbes, including pathogens. Flavohaemoglobins are arguably the most important, but not the only, mechanism by which the microbe strikes back.

Why then do higher organisms not also possess flavohaemoglobins? The answer may be that the high concentrations of other (non-flavo) globins protect animal cells from excessive NO; the combination of methaemoglobin reductase and very high haemoglobin concentrations in red blood cells provides an effective NO removal mechanism, functionally equivalent to the NO dioxygenase activities of flavohaemoglobin ^12^.

The term “nitrosative stress” appears to have been introduced to this field in 1996 ^13^ to describe specifically the reaction of S-nitrosothiols (RSNOs, such as S-nitrosocysteine) with intracellular thiols via S-nitrosation (that is, the transfer of the nitrosonium group NO ^+^ to biomolecules) ^4^. It is important to note that the term should not be used, as it sometimes appears to be, to describe all NO chemistry in biology: “NO cannot act as a nitrosating agent, unless there are oxidizing agents present, such as a transition metal species or oxygen. Thus, NO cannot nitrosate thiols. Reports to the contrary result from the presence, sometimes adventitiously, of an oxidizing agent or from an imprecise description of the reaction” ^4^. Thus, for example, the *Escherichia coli* flavohaemoglobin (Hmp), the subject of this article, cannot *directly* protect against nitrosating agents since only NO reacts in a physiologically useful way with this globin. “Nitrosative” is not an adjectival form of NO!

The broad reactivity of NO in biology implies that certain cellular components will be more susceptible to NO damage than others. A comprehensive kinetic model that encompasses this reactivity in *E. coli* that incorporates spontaneous and enzymatic reactions as well as damage and repair of biomolecules has been developed ^14^. This model, informed by experimental measurements of NO dynamics, allows a detailed analysis of how NO distributes in *E. coli* cultures and identification of the control parameters of the NO distribution. The simulation predicted that Hmp functions as a dominant NO consumption pathway at O _2_ concentrations as low as 35 μM (that is, microaerobic conditions): virtually all (99.85%) of the NO consumed by the cells was predicted to be through Hmp detoxification, and most of the remainder through oxidation by O _2_ and reaction with superoxide anion. Surprisingly, Hmp loses utility as the NO delivery rate increases, as a result of substrate inhibition ^15^. Such models are valuable for rigorously investigating NO stress in microbes and may identify novel strategies to potentiate the effects of NO ^14^.

## The discovery of the flavohaemoglobin Hmp

Hmp was discovered in bacteria in 1991 ^16^, only a year after key articles from Furgott, Ignarro and Murad identified the endothelium-derived relaxing factor (EDRF) as a gas with a molecular mass of only 30 and a year before the recognition of NO as “Molecule of the Year” by *Science* in 1992. Between 1989 and 1998, when the Nobel Prize for Physiology or Medicine was awarded to Furgott, Ignarro and Murad ^17– 19^, the citation count per annum increased almost 70-fold. In fact, the discovery that EDRF was NO was also made by Salvador Moncada, then at the Wellcome Research Laboratories in the UK, but, astonishingly, the Nobel award did not include Moncada. At the time, those involved in this work might not have foreseen how NO research would be so sustained (currently running at about 12,000 citations per annum) and all-encompassing in biology ( Figure 1), nor could we have known that the flavohaemoglobin Hmp would assume the role of the pre-eminent NO-detoxifying enzyme in microbes. However, other contenders exist (see below).

Hmp was not the first bacterial globin to be identified and sequenced. Rather, the first was the haemoglobin of *Vitreoscilla*, which is an obscure bacterium whose soluble haemoprotein (Vgb) is dramatically increased in concentration under the microaerobic conditions that the organism encounters ^20^. The function or functions of this protein are still unknown: despite evidence that its expression in heterologous hosts can confer some protection from nitrosative stress ^21^ and perhaps acts as an oxidase (although this is disputed ^22^), the generally accepted view is that Vgb facilitates oxygen utilisation; numerous articles have claimed biotechnological applications for this effect (see below). Vgb is unlike flavohaemoglobin: in a recent attempt to classify and logically name the globin family ^23, 24^, we proposed that one family should be the 3/3 myoglobin-like proteins. One sub-family comprises the two-domain flavohaemoglobins (having haem and reductase modules), and the second comprises the single-domain globins, of which the *Vitreoscilla* protein is one example that comprises only the haem domain.

Hmp was the first microbial globin for which a gene sequence was obtained, for which modes of regulation were established and, most importantly, for which a function was unequivocally demonstrated. The serendipitous discovery of the *hmp* gene by the author and colleagues ^16^ showed it to be a 44-kDa monomer with a haem domain that was almost half (46%) identical to Vgb. The C-terminal domain closely resembles ferredoxin-NADP ^+^ reductase in that both have highly conserved binding sites for NAD(P)H and FAD ^25^. Purified Hmp possesses haem B and FAD ^26, 27^, the presence of which was confirmed by the crystal structures of bacterial flavohaemoglobins ^28– 30^. This reductase domain, which transfers electrons from NAD(P)H to haem-bound ligands (and soluble molecules ^25, 31^), is essential for the function of Hmp ^32, 33^.

Here, the original names Hmp (intentionally and cautiously suggesting only a haemoprotein nature and not rashly assuming a function) and *hmp* are used for the protein and gene, respectively, in enterobacteria ^16^. Yhb in yeasts ^34^ and the more general abbreviation Fhb (flavohaemoglobin) are used elsewhere.

## The functions of flavohaemoglobin

**Figure 2.**
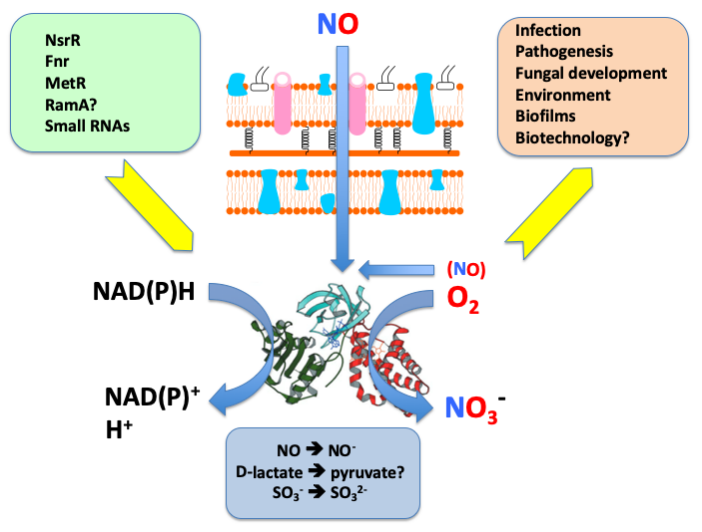
Flavohaemoglobin as a pre-eminent nitric oxide (NO)-detoxifying protein. A typical (Gram-negative) envelope is shown that allows ready access of extracellular NO to intracellular Hmp. A small contribution to the cellular NO pool from intracellular sources is indicated. Hmp comprises a haem domain (red), an NAD(P)H-oxidizing domain (green) and an FAD domain (cyan). The redox centres are shown. The primary reaction catalysed is the conversion, by a dioxygenase mechanism, of O _2_ and NO to form nitrate. Minor reactions also reported are shown below in the blue box. Transcriptional regulators identified thus far are shown at the right (green box), and the numerous consequences of Hmp activity are indicated at the right (orange box).

The first clue to function came from our discovery that solutions of NO gas (not a nitrosating agent) were potent inducers of *hmp* gene transcription ^35^. Shortly after, Gardner and colleagues demonstrated an enzymic function for Hmp, named NO dioxygenase (that is, the conversion of NO and O _2_ to innocuous NO _3_^−^) ^36^. An alternative interpretation of this critical inducible reaction is that Hmp is not a dioxygenase (that is, in which the two O atoms are used to oxygenate NO ^36, 37^) but a denitrosylase ^38, 39^; here, the haem-bound NO (Fe ^III^NO ^−^) reacts with an oxygen molecule to produce nitrate. However, later evaluation of the reaction mechanism confirmed the NO dioxygenase mechanism ^40^: analysis of the stoichiometric product (nitrate) showed more than 99% double O-atom incorporation from Hmp ^18^O _2_. The NO dioxygenation mechanism involves (1) rapid reaction of NO with a Fe ^III−^O _2_. intermediate (the product of the facile reaction of O _2_ with Hmp Fe ^II^ to form Fe ^III−^OONO) and (2) rapid isomerization of this intermediate to form nitrate. The O–O bond homolyzes to form a protein-caged [Fe ^IV^ = O .NO _2_] intermediate, and ferryl oxygen attacks .NO _2_ to form nitrate. This mechanism appears common to all higher haemoglobins and myoglobin that have been examined ^40, 41^ ( Figure 2).

Therefore, NO detoxification by Hmp is optimal when oxygen is abundant. If oxygen is low (0–50 μM), NO defences are severely compromised, exhibiting a roughly 30-fold increase in NO clearance time compared with anaerobic and aerobic conditions for the same addition of an NO donor compound (50 μM DPTA NONOate) ^42^. Modelling suggested that a steep drop in anoxic activity of NorV, a flavorubredoxin with NO removal activity, as [O _2_] fell, combined with impaired translational and Hmp activities at low [O _2_], results in suboptimal overlap of these two detoxification systems, resulting in up to a roughly 60% loss in their combined NO detoxification activities. In addition, at low [O _2_] conditions, the concentrations of NO and O _2_ oscillated, arising from kinetic competition for O _2_ between the aerobic respiratory oxidases and Hmp ^42^. Other candidates for NO detoxification are described later.

Although the NO dioxygenase activity of Hmp is the key mechanism for NO removal aerobically, an anoxic lower activity has been described ^43^. This exhibits a rate that is orders of magnitude slower than the O _2_-dependent reaction, and the physiological relevance is a matter of debate ^44^. Nevertheless, NO binds Fe(III) Hmp to generate a nitrosyl adduct that is stable anoxically but decays in air to reform the Fe(III) protein ^43^. NO displaces CO bound to Fe(II) Hmp but CO recombines after only 2 s at room temperature, indicative of NO reduction and dissociation from the haem. Direct demonstration by membrane-inlet mass spectrometry of NO consumption and nitrous oxide production during anoxic incubation of NADH-reduced Hmp confirm the reaction *in vitro* ^43^ ( Figure 2).

NO at nanomolar levels induces biofilm dispersal in numerous bacteria (for example, ^45, 46^). Consequently, NO-charged catheters have been investigated to prevent bacterial colonization ^47^. Thus, production of Fhb in *Pseudomonas aeruginosa* inhibits dispersal while imidazoles (see below) attenuate the prevention of dispersal ^48^.

Interestingly, rendering Hmp inactive in *Salmonella enterica* Gallinarum, in which RpoS and the SsrA/B regulator were also mutated, generated a hyper-susceptible strain that caused no mortality on injection into chickens. Vaccination of chickens with this strain conferred complete protection against challenge with virulent bacteria comparable to that achieved with a conventional vaccine strain ^49^. Disabling NO defences as a strategic utility is also suggested by the finding that elimination of ClpP (a major ATP-depended protease) largely eliminated NO detoxification by *E. coli* ^50^. The effect is due to deficient transcript levels of *hmp* and widespread perturbations in other NO-responsive genes.

Recently, a new function was proposed ( Figure 2): scavenging of the mild oxidant sulfur trioxide anion radical (STAR), a product in cells of (bi)sulfite oxidation ^51^. The reaction of STAR with ferrous globins is rapid, and STAR reacts 260- and 1000-fold faster with Ngb (neuroglobin) and Fhb, respectively, than with glutathione, suggesting a detoxification function. The Fhbs of yeast and bacteria exhibit this activity, and a flavohaemoglobin mutant of *Saccharomyces cerevisiae* was slow-growing in the presence of sulfide attributed to mitochondrial damage ^51^.

## Flavohaemoglobins are widely distributed in microorganisms

Although much of what we have learned about these globins has come from bacteria, predominantly *E. coli* (sequence, function, gene regulation, and three-dimensional structure), the first reports of a microbial globin were in yeast ^52– 55^. No involvement in NO chemistry was then suspected. Sequences for Fhbs are among the most numerous globin genes in bacteria of diverse taxons (533 sequences reported), exceeded only by class 2 truncated globins (622 sequences) ^24^. A newer survey identified 3318 Fhb sequences ^56^, comprising 2363 in bacteria and 204 in eukaryotes. Fhbs appear to be absent from Archaea ^57^. The bacterial sequences were distributed across 10 bacterial divisions with the highest number in the Proteobacteria. Interestingly, other divisions appear devoid of these proteins or they are uncommon, as in Bacteroidetes and Cyanobacteria.

Eukaryotic flavohaemoglobins are also found in protozoa, other fungi and two trypanosomes of insects. The protozoan parasite *Giardia intestinalis* possesses only five known haem proteins, one of which is flavohaemoglobin; this protein is expressed when trophozoites are exposed to NO or nitrite stresses and acts as an NO dioxygenase ^58, 59^. The key roles of flavohaemoglobins in NO homeostasis in filamentous fungi and yeast are now widely recognised ^60– 62^.

Both eukaryotic and bacterial flavohaemoglobins are generally considered soluble enzymes. Although bacterial flavohaemoglobins are normally recovered for purification from cytoplasmic fractions, around 30% are periplasmic in *E. coli* on the basis of Western immunoblotting ^63^, but the haem holoenzyme appears to be uniquely cytoplasmic. In yeast, Yhb is located in the cytosol, mitochondrial matrix and the intermembrane space but also in the inner membrane ^64^; however, the CO-binding fraction of Yhb is not present in inner membrane vesicles.

The manner as to how this important protein has become so widely distributed has recently been addressed ^23, 24, 56^. We proposed that the flavohaemoglobin gene family arose from an ancestral globin and later spread to eukaryotes via horizontal gene transfer ^23, 24^. Such transfers between the domains of life are infrequent in biology, but “single-protein metabolic modules” (for example, Fhb and its self-contained NO detoxification function) are prone to gene duplication (see below) and such horizontal gene transfer during evolution. A striking example of gene transfer from bacteria is afforded by a study of the acquisition by the eczema-causing fungus *Malassezia* of an Fhb from *Corynebacterium* and a concomitant increase in NO resistance ^56^.

## Physiological aspects

It is now accepted that Hmp detoxifies NO, primarily aerobically, supported by the following key observations, many of which are from the older literature; illustrative examples are given.

- Null *hmp* mutants of *Salmonella* and *E. coli* are hyper-sensitive to the antimicrobial activity of NO or *S*-nitrosoglutathione (GSNO) ^65– 69^.
- Hmp catalyses redox chemistry with NO and O _2_ at the haem, and the haem of Hmp is readily reducible by physiological substrate (NAD(P)H) by means of electron transfer from FAD ^31– 33, 70^.
- The level of Hmp correlates with the level of NO resistance of respiration in *E. coli*. Respiration of an *hmp* mutant is highly sensitive to sub-micromolar NO, whereas respiration in cells pre-induced by treatment with sodium nitroprusside (SNP) is resistant to NO concentrations up to 50 μM ^71^.
- Null *hmp* mutants of *Salmonella* and *E. coli* are hyper-sensitive to killing by human macrophages ^66, 72, 73^, and *hmp* mutants of *Yersinia pestis* are attenuated for virulence in the NO-rich infection bubo ^74^.
- Bacteria growing on the exterior of spleen microcolonies respond to soluble signals and induce synthesis of Hmp, thus eliminating inward NO diffusion and protection of interior bacterial population from NO-derived inducing signals ^75^.
- The Fhb of the plant pathogen *Erwinia chrysanthemi* confers NO tolerance on the fungus but also, by intercepting plant-derived NO, attenuates the hyper-sensitive response ^76^.
- *Salmonellae* experiencing nitrosative stress generate a burst of the alarmone nucleotide guanosine tetraphosphate (ppGpp). This activates transcription of valine biosynthetic genes, thereby re-establishing branched-chain amino acid biosynthesis that enables the translation of Hmp ^77^.
- The genome of the yeast *Candida albicans* contains three genes encoding flavohaemoglobin-related proteins but, based on studies of mutants lacking each of these genes, only one, CaYHB1, is responsible for NO consumption and detoxification ^78^. Loss of CaYHB1 increases the sensitivity of *C. albicans* to NO-mediated growth inhibition and decreases virulence in mice compared with that in wild-type strains.
- Duplicate flavohaemoglobins may have distinctive functions, and one has the established NO dioxygenase function. For example, a gene duplication event in the Actinobacteria is suggested to have given rise to a second clade of type II flavohaemoglobins with unusual structural and functional properties, including D-lactate metabolism ^79, 80^.
- Similarly, gene duplication seems to have generated fungal Fhb clades with different locations. In *Aspergillus oryzae* ^81^, Fhb1 is located in the cytosol and the clade 4 Fhb2 in mitochondria, so that mechanisms for NO depletion in each cellular compartment are effected.

## Regulation of flavohaemoglobin gene expression

NO or nitrosating agents up-regulate the *hmp* gene. In fact, *hmp* is consistently among the most highly up-regulated genes seen in genome-wide transcription profiling of *E. coli*, *Bacillus subtilis* and *Salmonella* cultures exposed to NO and nitrosating agents ^82– 84^, in *Salmonella* following infection and induction of NO synthesis in J774 cells ^73^, and in the plant symbiont *Sinorhizobium meliloti* ^85^. Recently, NO _2_, an air pollutant, was also reported to up-regulate *hmp* expression in *Pseudomonas* strains ^86^.

Although our understanding of *hmp* regulation is incomplete, several mechanisms have been identified and studied so far. These include several described in early studies ^87– 90^ and, more recently, a sigma-dependent small RNA ^91^. In *S. aureus*, the two-component regulator SrrAB, generally considered an oxygen sensor, regulates *hmp* under low-oxygen conditions or on exposure to NO ^92– 94^. In *Salmonella*, DksA recently emerged as an important factor for full expression of *hmp* transcription following NO exposure ^95^.

A major mechanism is undoubtedly via NsrR ^73, 96– 98^, a transcriptional repressor in the Rrf2 family containing an NO-sensitive FeS cluster (probably [4Fe-4S]). Reaction of the cluster with NO decreases its DNA-binding affinity and relieves repression at sensitive promoters that control expression of not only *hmp* but also *poxB* (via read-through from the upstream *hcp-hcr* genes) and the *sufABCDSE* cluster involved in iron-sulfur biogenesis and repair ^99, 100^. Recent work shows that *nsrR* is expressed from a strong promoter but that translation is inefficient. This is important since target promoters with low affinity for NsrR may partially escape repression ^99^. When H _2_O _2_ and NO coexist (as they do in the phagolysosome), NO detoxification is delayed, an effect attributed to inhibition by H _2_O _2_ of *hmp* gene transcription and translation under the control of NsrR ^101^.

In eukaryotic fungi such as *Aspergillus*, two proteins, FhbA and FhbB, are differentially induced to catabolise NO ^61, 102^. NO is produced endogenously by a nitrate reductase early in the transition from vegetative growth to development. NO homeostasis is critical since NO levels influence the balance between conidiation and sexual reproduction.

## Inhibitors of flavohaemoglobin activity and their utility

Since flavohaemoglobins confer a degree of pathogen resistance to NO generated by the immune system, including within the macrophage and its cocktail of reactive species (see above), inhibitors that target the haem prosthetic group of flavohaemoglobins are potential antimicrobial agents. Imidazoles having bulky aromatic substituents fit into the globin haem pocket and coordinate the ferric iron with a K _d_ of 333 μM ^30^. Structural studies confirm that azole binds the Fhb haem and reveal major conformational reorganisation ^103, 104^.

Others (miconazole, econazole, clotrimazole and ketoconazole) have similar activities against Fhbs ^105^ and inhibit NO metabolism in bacteria and yeast. However, they do not achieve the NO-induced stasis seen in flavohaemoglobin-null mutants. One of these agents, miconazole, is the most effective azole against *Staphylococcus* and ligates to both ferric and ferrous globin ^106^. Over 20 years ago ^107, 108^, we reported that, in the absence of NO, Hmp generates superoxide anion by single electron transfer from NAD(P)H to haem-bound oxygen. Interestingly, miconazole enhanced superoxide production by the *S. aureus* enzyme, so that, in macrophages, bacteria possessing flavohaemoglobin are compromised in survival compared with flavohaemoglobin-deficient bacteria ^106^. This presumably is attributed to the inhibitor binding to haem and diverting electrons to oxygen ^107, 108^. Other acceptors ^31^ may also be reduced when haem function is blocked, as occurs with CO ^31^.

Alternative inhibitors might be found among quinones and nitroaromatic compounds. *S. aureus* flavohaemoglobin rapidly reduces these compounds, which may act as subversive substrates, diverting electron flux from FAD and enhancing the toxicity of NO by formation of superoxide ^109^.

## Flavohaemoglobins in biotechnology?

There have been countless reports of the ability of the *Vitreoscilla* globin, when expressed in heterologous hosts, to enhance aerobic cell yields or product formation ^110– 112^. The mechanistic basis of these diverse effects remains unclear. However, a recent report ^113^ claims that both an exogenously introduced *Vitreoscilla* globin and the native Fhb enhance pullulan production in the yeast *Aureobasidium*. Fhb gene expression in the yeast was elevated 3.5-fold over native levels, based on reverse transcription polymerase chain reaction (RT-PCR) data, but CO difference spectra show only a modest increase in a pigment with some of the characteristics of globins or other haem proteins; the broad absorbance spectra and noisy traces should not be interpreted as unequivocal evidence of high globin expression. Expression of either globin increased oxygen uptake, but the data are not quantified and no mechanism for enhanced pullulan yields is presented.

*E. coli* Hmp, anchored to electrodes, electrolytically interconverts NADH and NAD ^+^ by transfer of electrons to the FAD moiety where NADH/NAD ^+^ is transformed. It is suggested that this might be employed in NAD-dependent bioelectrodes for biosyntheses, biosensors and biofuel cells ^114^.

## Flavohaemoglobin is not unique as a nitric oxide–detoxifying machine

Many other globins convert NO to NO _3_^−^ via an NO dioxygenase activity. These include the truncated globin of *Mycobacterium tuberculosis*, trHbN ^115^, the single-domain globin Cgb of *Campylobacter jejuni* ^116^, *M. tuberculosis* HbN ^115^, mammalian cytoglobin ^117^ and *Arabidopsis* cytoglobin 3 ^118^.

When oxygen is available, the catalytic efficiency of the Fhb reaction, k _cat_/K _m_, is very high: up to 2400 × 10 ^6^ M ^−1^ s ^−1^ ^119^. However, under anoxic or low-oxygen conditions, where the activity of Hmp is dramatically reduced, NO tolerance in *Salmonella* is affected additionally by a combination of three enzymes, flavorubredoxin (NorV), and cytochrome *c* nitrite reductase (NrfA). A study of the effects of all eight possible combinations of *norV*, *hmp* and *nrfA* single, double and triple mutations suggested an important additive role for both NorV and NrfA ^120, 121^. None of the NO detoxification systems—Hmp, NorV and NrfA—is solely responsible for nitrosative stress tolerance of *S. typhimurium* in raw sausages where sodium nitrite is used as a curing agent ^122^. Somewhat different conclusions were reached in a study of a uropathopathogenic strain of *E. coli* (UPEC) that induces a variety of defence mechanisms in response to NO, including direct NO detoxification (Hmp, NorVW, NrfA), iron-sulfur cluster repair (YtfE), and the expression of the NO-tolerant cytochrome *bd*-I respiratory oxidase (CydAB) ^123, 124^. During UPEC growth and survival during infection, loss of the flavohaemoglobin Hmp and cytochrome *bd*-I elicited the greatest sensitivity to NO-mediated growth inhibition, whereas all but the periplasmic nitrite reductase NrfA provided protection against neutrophil killing and promoted survival within activated macrophages. Intriguingly, cytochrome *bd-*I was the only system that augmented UPEC survival in a mouse model, suggesting that maintaining aerobic respiration under conditions of nitrosative stress is a key factor for host colonisation. In *Salmonella enterica* also, cytochrome *bd* augments defences against NO in systemic tissues ^125^. Thus, cytochrome *bd* emerges as a major contributor to bacterial NO tolerance and host colonisation under microaerobic conditions. The hybrid cluster protein Hcp and its NADH-dependent cognate reductase Hcr were not tested in this study, but it is striking that a role for this system as a high-affinity NO reductase could be demonstrated only when the Hcp reductase was introduced into a strain deleted for the *nirBD*, *nrfAB*, *norVW*, *hmp* and *hcp* genes ^126^. Other non-globin contenders include the flavorubredoxin and nitrite reductase NrfA of *E. coli* ^127^, various flavodiiron proteins, *Paracoccus denitrificans* NO reductase NorBC, and the NO reductase of *S. aureus* ^128^.

## Conclusions and outlook

The flavohaemoglobins of numerous bacterial species and groups, yeasts, fungi and protozoa continue to fascinate those devoted to understanding globin functions, NO homeostasis in biology and clinical medicine. Our knowledge has exploded since their discovery in 1991 (at least at a molecular level) and has revealed new paradigms of enzyme mechanisms, gene regulatory mechanisms and physiological significance. Undoubtedly, much remains to be learned. It is striking, though, that throughout this period (almost 30 years), no clear evidence has emerged for a flavohaemoglobin in higher organisms. This continues to offer the hope that such a protein, a “single protein metabolic module”, might represent a useful target for antimicrobial therapies. Imidazoles with great efficacy have been identified as inhibitors and these efforts, in concert with increasing understanding of protein function, ligand and electron migration within such flavoproteins, may yet give us new antimicrobial weapons.
